# Admission serum tropomyosin 4 levels predict 1-year functional outcomes in acute ischemic stroke

**DOI:** 10.7717/peerj.20745

**Published:** 2026-02-04

**Authors:** Keying Wu, Mingxi Chen, Huan Wang, Yuyi Zhu, Yaqi Chen, Shihong Zhang, Xinyi Leng, Zilong Hao, Deren Wang

**Affiliations:** 1 Department of Neurology and Center of Cerebrovascular Diseases, West China Hospital, Sichuan University, Chengdu, Sichuan, China; 2 Department of Medicine and Therapeutics, The Chinese University of Hong Kong, Hong Kong SAR, China; 3 Department of General Medicine, West China Le Cheng Hospital, Sichuan University, Qionghai, Hainan, China; 4 Microbiology and Intelligent Biomanufacturing Key Laboratory of Sichuan Province, College of Life Science, Sichuan University, Chengdu, Sichuan, China

**Keywords:** Tropomyosin 4, Ischemic stroke, Biomarker, Prognostic prediction, Biomarker-based prediction models

## Abstract

**Background:**

Tropomyosin 4 (TPM4) regulates neurite outgrowth and vascular pathology but its role as a biomarker for predicting outcomes in stroke patients is unclear. This study investigated the association between serum TPM4 levels and 1-year functional outcomes in acute ischemic stroke (AIS) patients.

**Methods:**

AIS patients admitted within 24 h post-onset from the Chengdu Stroke Registry were included. Serum TPM4 levels were measured by enzyme-linked immunosorbent assay (ELISA). Poor functional outcomes were defined as a modified Rankin Scale (mRS) score >2 at 1 year after stroke onset. Multivariate logistic regression assessed TPM4’s association with outcomes, with its predictive incremental value evaluated by discrimination, reclassification, and overall performance metrics.

**Results:**

Among 181 patients (median age 66 years, 64.1% male), 59 (32.6%) experienced poor outcomes at 1 year, including 16 deaths (8.8%). Serum TPM4 levels on admission were negatively correlated with the National Institutes of Health Stroke Scale (NIHSS) score (*r* = −0.185, *p* = 0.013). Adjusted for confounders, lower serum TPM4 levels were independently associated with 1-year poor functional outcomes (adjusted OR 0.045, 95% CI [0.005–0.393], *p* = 0.005). Serum TPM4 levels had acceptable discriminative ability for predicting poor outcomes (AUROC 0.706, 95% CI [0.621–0.791], *p* < 0.0001). Incorporating TPM4 into the basic model significantly improved the predictive power for poor functional outcomes (net reclassification index: 31.87%, *p* = 0.041; integrated discrimination improvement: 5.01%, *p* = 0.008; Brier score decreased from 0.16 to 0.15, *p* = 0.012).

**Conclusions:**

Lower serum TPM4 levels on admission were independently associated with poor functional outcomes at 1 year in AIS patients, suggesting that TPM4 may serve as a potential biomarker for long-term outcomes and offer insights into its potential role in stroke pathophysiology. These findings need to be further verified in external cohorts.

## Introduction

Acute ischemic stroke (AIS) is a leading cause of disability and mortality worldwide (Feigin et al., 2021). Early risk stratification of patients at high risk of poor outcomes is crucial for optimizing treatment and rehabilitation. However, conventional clinical predictors have limited value for long-term prognosis (Hillen et al., 2003). Therefore, novel blood biomarkers are needed to improve prognostic prediction and provide insights into the complex pathophysiology of ischemic stroke (Tiedt et al., 2018; Montellano et al., 2021; Meisinger et al., 2024).

Cytoskeletal disruption is a key feature of ischemic neuronal injury, contributing to loss of protein transport and structural stability (Dewar & Dawson, 1997). Proteins associated with the actin cytoskeleton support neurovascular unit survival and post-ischemic repair (Kühn, Meissner & Oehmichen, 2005; Posada-Duque, Barreto & Cardona-Gomez, 2014). Tropomyosin 4 (TPM4), an actin-binding protein that regulates actin-myosin interactions, is widely expressed in neurons and involved in neurite growth and synaptic plasticity (Phillips et al., 1979; Guven, Gunning & Fath, 2011; Had et al., 1994; Curthoys et al., 2014; Gray, Kostyukova & Fath, 2017; Genoud et al., 2025). Reduced tropomyosin expression in the ischemic penumbra during the hyperacute phase of cortical infarction suggests its potential role as a marker of neuronal injury (Demyanenko & Uzdensky, 2017; Yu et al., 2023).

TPM4 is also implicated in vascular pathology. It is upregulated in smooth muscle cells during atherosclerosis and associated with coronary artery calcification and plaque stability (Abouhamed et al., 2003; Sung et al., 2021; Gulsin & Moss, 2021; Zhang et al., 2022; Dardé et al., 2010). Moreover, anti-TPM4 antibodies have been linked to cerebral small vessel disease through endothelial dysfunction (Kimura et al., 2012).

Despite evidence implicating TPM4 in both neuronal integrity and vascular pathophysiology, the prognostic value of TPM4 in AIS remains unknown. This study investigates the association between admission serum TPM4 levels and 1-year functional outcomes after AIS, aiming to evaluate its potential as a prognostic biomarker.

## Materials and Methods

### Study population

We retrospectively analyzed data from a consecutive series of AIS patients admitted to the Department of Neurology at West China Hospital, Sichuan University, between December 2018 and December 2021. All patients were prospectively enrolled in the Chengdu Stroke Registry (Liu et al., 2019; Wang et al., 2024). This study was approved by the Ethics Committee on Biomedical Research at West China Hospital, Sichuan University (Ethical Application Ref: 2023 [482]). Written informed consent was obtained from patients or their authorized representatives. This study is reported in accordance with ‘Standards for Reporting Diagnostic Accuracy Studies’ (STARD) guidelines (Bossuyt et al., 2015).

The inclusion criteria of this study were: (1) age ≥18 years, (2) admission within 24 h of stroke onset, (3) confirmed ischemic stroke diagnosis based on computed tomography (CT) or magnetic resonance imaging (MRI), (4) available blood samples for serum TPM4 measurements, and (5) modified Rankin Scale (mRS) score of 0–2 prior to stroke onset. According to the World Health Organization (WHO) criteria, ischemic stroke refers to the acute focal neurological dysfunction caused by single or multiple infarctions in the brain or retina that lasts over 24 h or shows evidence of acute infarction in clinically relevant brain areas based on neuroimaging or other techniques (WHO, 2023). Exclusion criteria included: (1) malignant tumors, (2) severe liver disease and/or end-stage kidney disease, (3) heart failure, including acute coronary syndrome during hospitalization, (4) serious infections, (5) concurrent autoimmune diseases, and (6) incomplete 1-year follow-up.

### Baseline data collection

Baseline characteristics, including demographic information, clinical features, vascular risk factors, NIHSS score at admission, Trial of Org 10172 in Acute Stroke Treatment (TOAST) classification, and information on reperfusion therapy, were extracted from the Chengdu Stroke Registry.

### Measurement of serum TPM4 levels

Peripheral blood samples were collected within 48 h of the patient’s arrival at the Emergency Department. Serum was isolated by centrifugation and immediately stored at −80 °C. Serum TPM4 concentrations were measured using a commercial enzyme-linked immunosorbent assay (ELISA) kit (MB-1508A, Jiangsu Meibiao Biotechnology Co., Ltd., Jiangsu, China). The assays were conducted by laboratory technicians blinded to outcome data at K J Biotechnology Co., Ltd., Sichuan, China.

### Clinical outcome

Functional outcomes were assessed at 1-year follow-up using the mRS. A score >2 was classified as a poor functional outcome (Hill et al., 2020). Standardized telephone interviews were conducted by trained vascular neurologists to determine mRS scores.

### Statistical analysis

Categorical variables were summarized as numbers and percentages and compared using the chi-squared test. Continuous variables were tested for normality using the Shapiro-Wilk test and found to have a skewed distribution. Therefore, they are presented as medians with interquartile ranges (IQR) and compared using the Mann-Whitney U test. Stroke severity, classified as mild (NIHSS ≤5), moderate (6–15), and severe (>15), was analyzed using the Kruskal-Wallis test with Bonferroni corrections for multiple comparisons (Adams et al., 1999; Meng et al., 2024). Spearman’s rank correlation was used to analyze the association between serum TPM4 levels and NIHSS scores. A receiver operating characteristic (ROC) curve was generated to identify the optimal serum TPM4 cutoff value for predicting poor outcomes.

Due to limited statistical power, parsimonious multivariate logistic regression was performed to assess the association between serum TPM4 levels and poor outcomes, adjusting for variables that have been identified as the most important covariates (age, NIHSS score at admission, reperfusion therapy) for poor outcome after ischemic stroke in previous studies according to current statistical guidance (Heinze, Wallisch & Dunkler, 2018). As the parameter investigated had a non-Gaussian distribution, serum TPM4 levels were natural log-transformed or dichotomized using the ROC-derived cutoff value for inclusion in the regression model. *Post-hoc* power analysis was conducted using Wald test-based calculations and bootstrap validation with 100 resamples (Steyerberg et al., 2001; Moons et al., 2012; Demidenko, 2007). Subgroup analyses were conducted to evaluate potential effect modifications by age, sex, baseline NIHSS score, atrial fibrillation, TOAST classification, and reperfusion therapy.

To further estimate the incremental value of serum TPM4 to the conventional risk factors, two logistic regression models were constructed: a basic model incorporating conventional risk factors (age, NIHSS score at admission, reperfusion therapy), and a new model that included serum TPM4 alongside these risk factors. Area under the Receiver Operating Characteristic Curve (AUROC) analysis and DeLong test were performed to compare the discriminative performance of the two models for predicting poor functional outcomes. The net reclassification index (NRI) and integrated discrimination improvement (IDI) were calculated to assess the improvement in risk reclassification provided by the new model compared to the basic model (Pencina et al., 2008). The overall model performance was assessed using the Brier score (the lower the better) (Steyerberg et al., 2010). Odds ratios (ORs) were reported with 95% confidence intervals (95% CI). A *p*-value < 0.05 was considered statistically significant. Data analysis was performed using IBM SPSS Statistics (version 26.0; IBM, Armonk, NY, USA), GraphPad Prism (version 9.1; GraphPad Software, San Diego, CA, USA), and R (version 4.3.1 for Windows).

## Results

### Patients included *vs*. excluded

**Figure 1:**
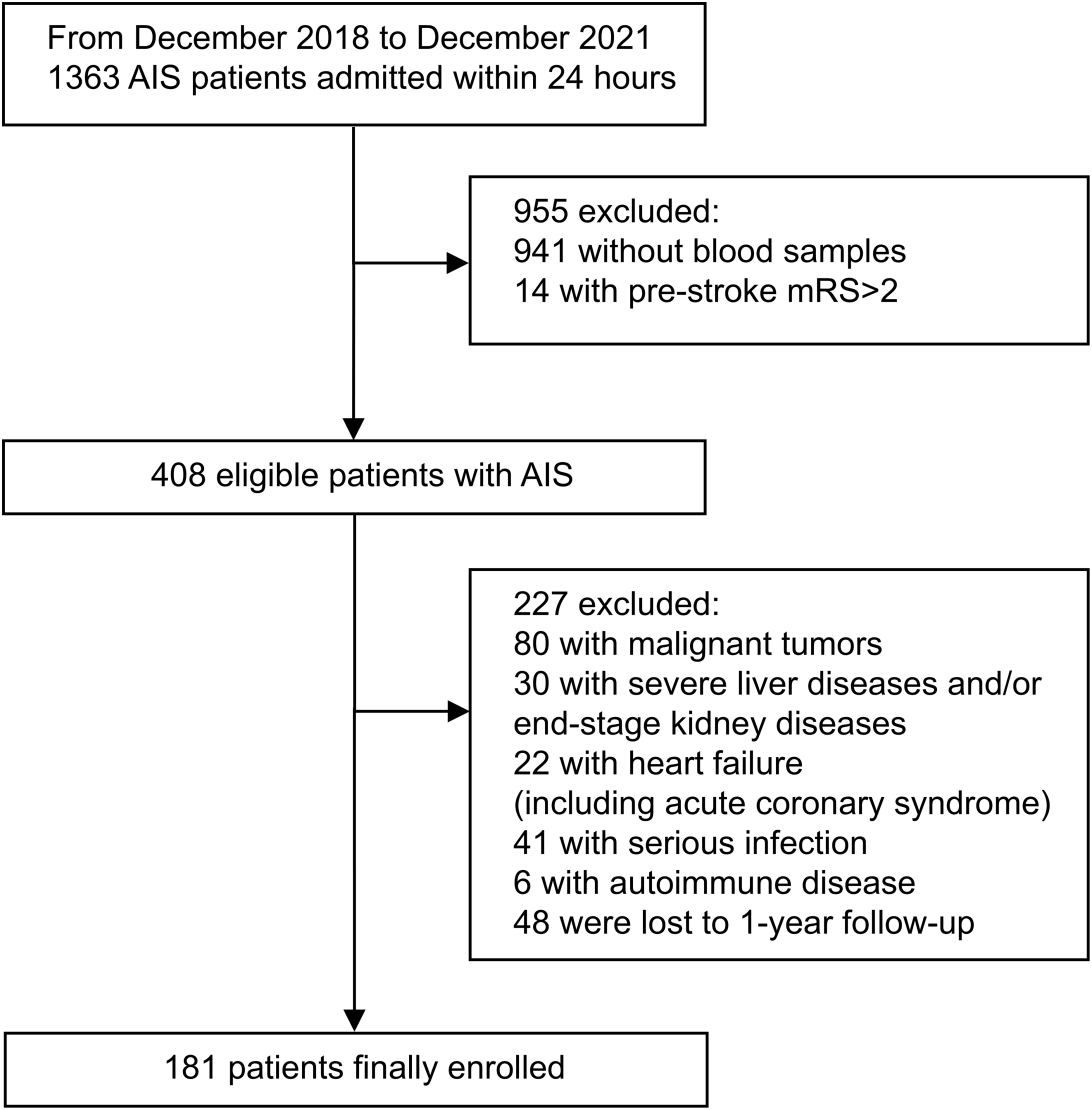
Flow chart for patient screening process.

From an initial cohort of 1,363 AIS patients admitted within 24 h of symptom onset, 181 were included in the final analysis (Fig. 1). Baseline characteristics were generally comparable between included and excluded patients, except for differences in stroke onset-to-admission interval (5.0 (IQR 3.0–10.0) *vs*. 5.0 (IQR 3.0–13.3); *p* = 0.023) and TOAST classification (*p* = 0.014) (Table S1, Supplemental Files). As well, the 48 patients lost to follow-up were similar to those included except for baseline NIHSS score (8 (IQR 3–14) *vs*. 16 (IQR 4–20); *p* = 0.002) (Table S2, Supplemental Files).

### Baseline characteristics of the cohort included

Among the 181 patients who were included, the median age was 66 years (IQR 55–72), with 64.1% male. The median NIHSS score at admission was 8 (IQR 3–14). According to TOAST, 45.3% of patients (*n* = 82) had large artery atherosclerosis (LAA) stroke, while 29.8% (*n* = 54) had cardioembolic (CE) stroke. 25.4% of patients (*n* = 46) received endovascular therapy (EVT) alone, 11.0% (*n* = 20) received intravenous thrombolysis (IVT) alone, and 6.1% (*n* = 11) received bridging therapy. At 1-year follow-up, 32.6% (*n* = 59) of patients experienced poor functional outcomes. Patients with poor outcomes were significantly older [69 (IQR 64–74) *vs*. 63 (IQR 53–70); *p* = 0.023], included a lower proportion of males (50.8% *vs*. 70.5%, *p* = 0.010), and had a higher baseline NIHSS score [14 (IQR 9–17) *vs*. 4 (IQR 2–11); *p* < 0.001] and a higher proportion of atrial fibrillation (AF) (50.8% *vs*. 19.7%, *p* < 0.001) (Table 1).

**Table 1:** Baseline characteristics based on 1 year function outcome.

Variables	Total	Good outcome (mRS score 0–2)	Poor outcome (mRS score 3–6)	*p* value
No. (%)	181 (100)	122 (67.4)	59 (32.6)	
Demographics				
Age, years	66 (55–72)	63 (53–70)	69 (64–74)	<0.001*
Male, *n* (%)	116 (64.1)	86 (70.5)	30 (50.8)	0.010*
Clinical features				
Onset-to-admission interval, hours	5.0 (3.0–10.0)	5.0 (3.0–9.3)	5.0 (3.0–10.0)	0.956
NIHSS	8 (3–14)	4 (2–11)	14 (9–17)	<0.001*
Vascular risk factors, *n* (%)				
Hypertension	111 (61.3)	77 (63.1)	34 (57.6)	0.477
Diabetes mellitus	49 (27.1)	33 (27.0)	16 (27.1)	0.992
Hyperlipidemia	23 (12.7)	17 (13.9)	6 (10.2)	0.476
Coronary artery disease	20 (11.0)	14 (11.5)	6 (10.2)	0.793
Atrial fibrillation	54 (29.8)	24 (19.7)	30 (50.8)	<0.001*
Stroke history	19 (10.5)	12 (9.8)	7 (11.9)	0.676
TOAST classification				<0.001*
LAA	82 (45.3)	56 (45.9)	26 (44.1)	
CE	54 (29.8)	25 (20.5)	29 (49.2)	
SAO	28 (15.5)	26 (21.3)	2 (3.4)	
SOC	5 (2.8)	4 (3.3)	1 (1.7)	
SUC	12 (6.6)	11 (9.0)	1 (1.7)	
Reperfusion therapy				0.073
No	104 (57.5)	76 (62.3)	28 (47.5)	
IVT	20 (11.0)	15 (12.3)	5 (8.5)	
EVT	46 (25.4)	24 (19.7)	22 (37.3)	
Bridge treatment	11 (6.1)	7 (5.7)	4 (6.8)	
TPM4, ng/mL	2,432.9 (2,216.1–2701.4)	2,511.4 (2,284.6–2,781.1)	2,252.2 (2,050.7–2,522.2)	<0.001*

**Notes:**

Abbreviations: mRS, modified Rankin Scale; NIHSS, National Institute of Health Stroke Scale; TOAST, Trial of Org 10172 in Acute Stroke Treatment; LAA, large artery atherosclerosis; CE, cardioembolism; SAO, small artery occlusion; SOC, stroke of other determined cause; SUC, stroke of undetermined cause; IVT, intravenous thrombolysis; EVT, endovascular therapy; TPM4, Tropomyosin 4.

* *p* < 0.05.

### Correlation between TPM4 levels and stroke severity

**Figure 2:**
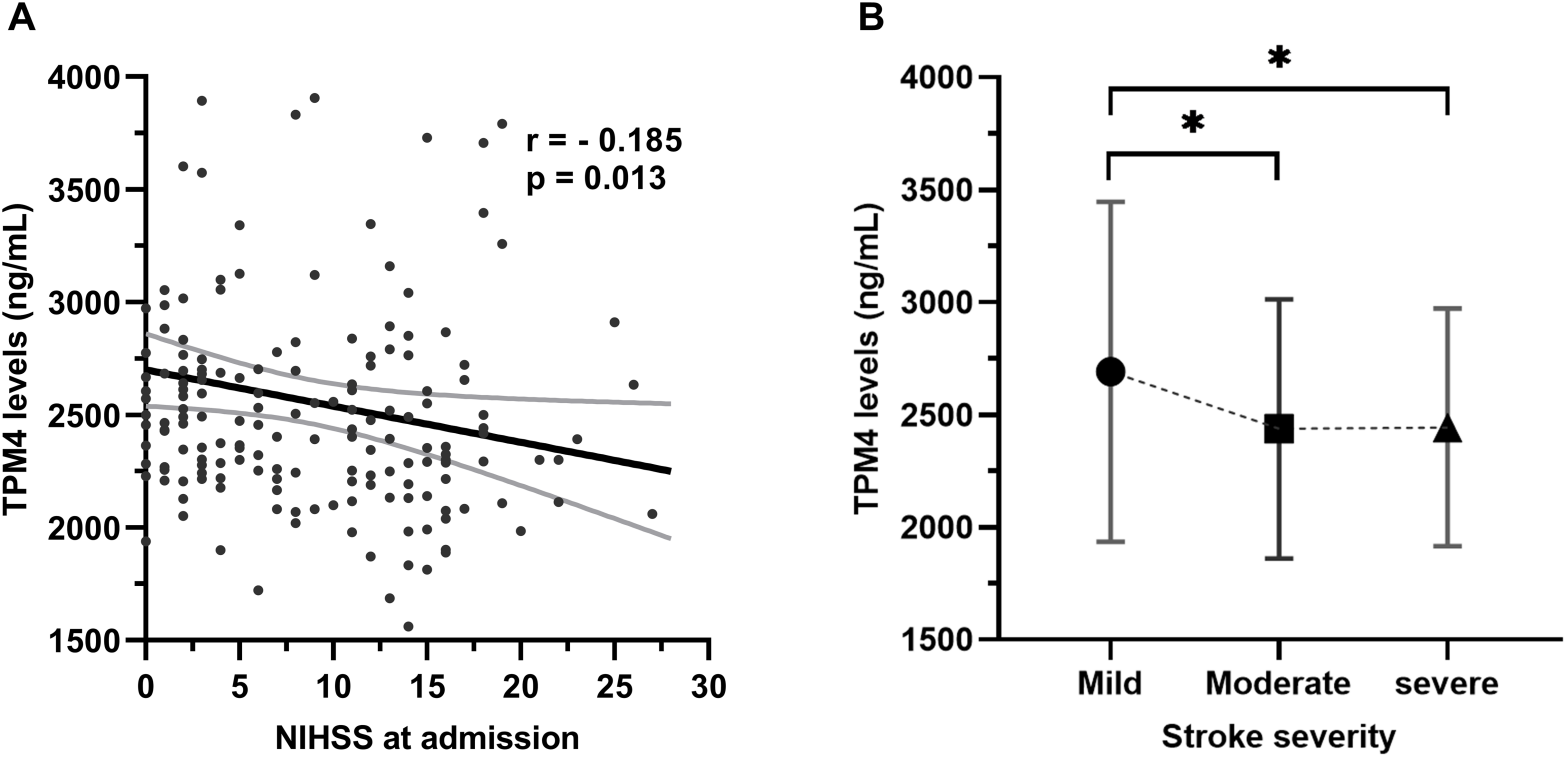
Association with serum TPM4 levels and NIHSS at admission. (A) Spearman’s correlations between NIHSS on admission and serum TPM4 levels. (B) Comparison of TPM4 levels among patients stratified by stroke severity (NIHSS scores). TPM4 levels in the three groups were as follows: mild group (2,509.4 [2,285.0–2,751.5] ng/mL), moderate group (2,393.1 [2,134.5–2,700.7] ng/mL), and severe group (2,300.0 [2,082.8–2,655.0] ng/mL). *P < 0.05.

At admission, there was a significant inverse correlation between serum TPM4 levels and NIHSS scores (r = −0.185, *p* = 0.013; Fig. 2A). Patients who had mild strokes had the highest levels of TPM4, followed by those who had moderate and severe strokes (Fig. 2B).

### Association between TPM4 levels and stroke outcomes

Patients with poor functional outcomes had significantly lower serum TPM4 levels compared to those with good outcomes at 1 year [2,252.2 ng/mL (IQR 2,050.7–2,522.2) *vs*. 2,511.4 ng/mL (IQR 2,284.6–2,781.1); *p* < 0.001] (Table 1). After adjusting for age, baseline NIHSS score and reperfusion therapy, lower serum TPM4 levels at admission were independently associated with poor outcomes at 1 year (adjusted OR 0.045 per e-fold increase in TPM4, 95% CI [0.005–0.393], *p* = 0.005; Table 2). *Post-hoc* statistical power was 81.1% by Wald test and 85.0% by bootstrap validation, both exceeding the conventional 80% threshold.

**Table 2:** Logistic regression adjusted for patients’ age, NIHSS score at admission, reperfusion therapy.

Variables	Adjusted OR	95% CI	*p* value
Age	1.041	[1.007–1.077]	0.019*
NIHSS	1.178	[1.099–1.264]	<0.001*
Reperfusion therapy			0.902
No	NA	NA	Ref
IVT	0.694	[0.185–2.610]	0.589
EVT	1.025	[0.406–2.591]	0.958
Bridge treatment	0.674	[0.145–3.129]	0.651
TPM4^a^	0.045	[0.005–0.393]	0.005*

**Notes:**

a The concentration of TMP4 was natural ln-transformed before fitting the logistic regression model.

Abbreviations: OR, odds ratio; CI, confidence interval; NA, not applicable; Ref, reference. NIHSS, National Institute of Health Stroke Scale; IVT, intravenous thrombolysis; EVT, endovascular therapy; TPM4, Tropomyosin 4; Ln, natural logarithm.

* *p* < 0.05.

**Figure 3:**
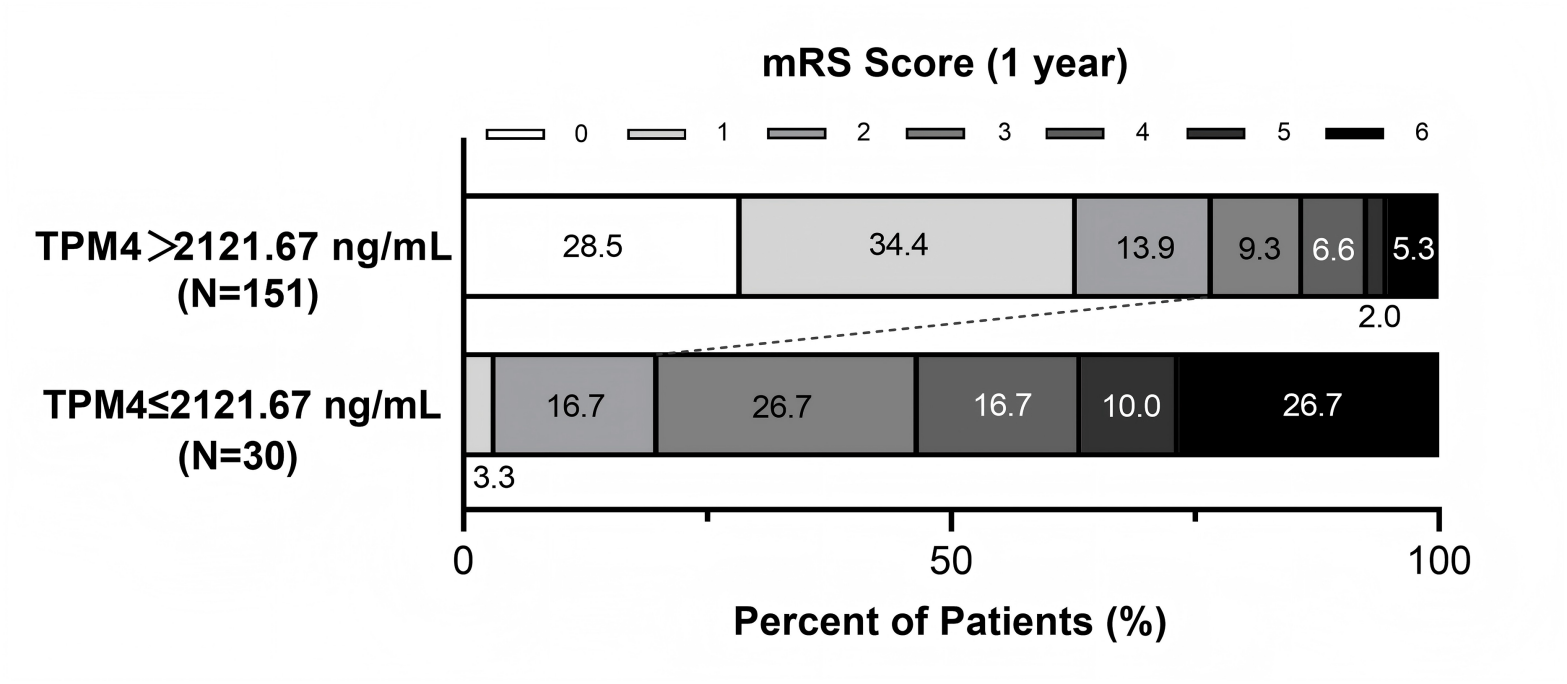
mRS score for patients with higher TPM4 level *vs*. those with lower TPM4 level at 1 year after stroke. The cutoff value of the TPM4 level for outcome is established through ROC curves.

ROC analysis demonstrated that serum TPM4 levels had acceptable discriminative ability for predicting poor outcomes (AUROC 0.706, 95% CI [0.621–0.791], *p* < 0.0001; Fig. S1, Supplemental Files). Using an optimal cutoff value of 2,121.67 ng/mL derived from the ROC curve, patients with lower TPM4 were more likely to experience poor functional outcomes (Fig. 3). Multivariate analysis confirmed serum TPM4 remained an independent predictor when treated as a dichotomous variable (adjusted OR 0.115, 95% CI [0.039–0.338], *p* < 0.001; Table S3, Supplemental Files).

### Subgroup analysis

Subgroup analyses stratified by age, sex, baseline NIHSS score, AF, TOAST classification, and reperfusion therapy consistently showed that higher serum TPM4 levels were associated with a lower risk of poor outcomes. No significant interactions between TPM4 levels and these factors were observed (all *p* for interaction > 0.05; Table S4, Supplemental Files).

### Incremental value of TPM4 for outcome prediction

Compared with the basic model, the new model incorporating serum TPM4 showed a numerically higher discrimination, with the AUROC increasing from 0.824 (95% CI [0.762–0.886]) to 0.845 (95% CI [0.784–0.905]), although this improvement was not statistically significant (*p* = 0.228 by DeLong’s test). Notably, the addition of serum TPM4 significantly improved reclassification for poor functional outcomes [NRI: 31.87% (95% CI [1.35–62.39%], *p* = 0.041); IDI: 5.01% (95% CI [1.30–8.72%], *p* = 0.008)] and enhanced overall performance, with a decreased Brier score from 0.16 (95% CI [0.13–0.19]) to 0.15 (95% CI [0.12–0.18], *p* = 0.012) (Table 3).

**Table 3:** Incremental predictive value of serum TPM4 for functional outcomes in AIS.

Model	Continuous NRI		IDI		Brier score	
	Estimate (95%CI), %	*p* value	Estimate (95%CI), %	*p* value	Estimate (95%CI)	*p* value
Basic model	–	Ref	–	Ref	0.16 [0.13–0.19]	Ref
Basic model +TPM4	31.87 [1.35–62.39]	0.041*	5.01 [1.30–8.72]	0.008*	0.15 [0.12–0.18]	0.012*

**Notes:**

Basic model for poor functional outcomes at 1 year adjusted for age, National Institutes of Health Stroke Scale and reperfusion therapy.

Abbreviations: NRI, net reclassification index; IDI, integrated discrimination improvement; TPM4, Tropomyosin 4; Ref, reference; CI, confidence interval.

* *p* < 0.05.

## Discussion

Blood biomarkers are emerging tools for individualized outcome prediction, facilitating early intervention and personalized rehabilitation in stroke care. This study provides the first clinical evidence linking serum TPM4 levels with long-term functional outcomes after AIS, identifying TPM4 as an independent prognostic biomarker. Serum TPM4 levels were also negatively correlated with stroke severity. Moreover, TPM4 improved prognostic model performance through better reclassification (NRI, IDI) and overall accuracy (Brier score), underscoring its potential clinical utility.

Experimental data support the biological plausibility of these findings. Previous studies have shown that tropomyosin expression is down-regulated in the cerebral cortex after focal photothrombotic infarction (PTI), indicating cerebral tissue destruction (Dewar & Dawson, 1997; Demyanenko & Uzdensky, 2017). Another study showed that TPM4 expression gradually increased after reperfusion in a mouse middle cerebral artery occlusion (MCAO) model and peaked at 12 h (Yu et al., 2023). However, no clinical data have previously investigated the relationship between TPM4 and stroke outcomes. In this study, lower admission TPM4 levels were associated with more severe strokes and poorer 1-year outcomes. Taken together with previous and our findings, TPM4 levels may reflect the extent of initial injury and the capacity for long-term functional recovery after AIS, potentially acting as a neuroprotective factor.

The mechanisms underlying TPM4’s neuroprotective role are not yet clear but likely involve cytoskeletal stabilization and synaptic remodeling. During the subacute phase of stroke, neuronal plasticity—including synapse formation and circuit reorganization—supports recovery. TPM4 stabilizes actin filaments and regulates ADF/cofilin phosphorylation, promoting synaptic formation and long-term potentiation *via* the Neuroligin-1–LIMK1–ADF/cofilin pathway (Bryce et al., 2003; Brettle, Patel & Fath, 2016). Overexpression of TPM4 enhances neurite outgrowth, growth cone formation, and dendritic arborization in B35 neuroblastoma cells and cultured hippocampal neurons (Gray, Kostyukova & Fath, 2017; Curthoys et al., 2014; Häbig et al., 2013). Furthermore, TPM4-mediated suppression of Sushi domain-containing protein 2 (SUSD2) may facilitate dendritic remodeling and axonal sprouting (Brettle, Patel & Fath, 2016; Sheng & Chen, 2020; Nadjar et al., 2015; Chamak & Prochiantz, 1989; Dickson, 2002). Collectively, TPM4 may contribute to post-ischemic neural repair by preserving the neurovascular unit and supporting synaptic plasticity, though further mechanistic research is warranted.

Evaluating the incremental prognostic value of emerging biomarkers is key for precision stroke care. Adding serum TPM4 to established clinical variables improved risk reclassification and overall predictive performance, even though the AUC increase did not reach statistical significance. These results indicate that TPM4 may help identify AIS patients at higher risk of long-term functional impairment or mortality, guiding more intensive monitoring and targeted rehabilitation.

Several limitations should be acknowledged. First, TPM4 was measured at a single time point; serial measurements could better capture its temporal dynamics during the acute and subacute phases. Second, comorbidities and medications, such as hypertension, diabetes, or statin therapy, may influence TPM4 expression and confound the results. Third, although baseline characteristics were generally comparable, this was a retrospective analysis of a single-center cohort with a limited sample size, which may introduce selection bias. Finally, stroke location and large-vessel occlusion status were not fully adjusted for and may have affected outcomes. Future large, multicenter studies with repeated biomarker measurements are needed to validate TPM4 as a reliable prognostic indicator.

## Conclusion

This study demonstrates that lower serum TPM4 levels at admission are associated with greater stroke severity and poorer 1-year functional outcomes in AIS patients. TPM4 provides incremental prognostic value beyond conventional predictors and may reflect neuroprotective and plasticity-related processes in stroke recovery. Further validation in larger cohorts and mechanistic studies is needed to confirm its clinical relevance and potential therapeutic implications.

## Supplemental Information

10.7717/peerj.20745/supp-1 Supplemental Information 1 Raw Data. The baseline characteristic data of the included and excluded patients, as well as the serum TPM4 concentration and functional outcome data of the patients included in the final analysis.

10.7717/peerj.20745/supp-2 Supplemental Information 2 Receiver operating characteristic curve analysis of serum TPM4 and poor outcomes of patients with AIS. Abbreviations: AUC, area under the curve; AIS, acute ischemic stroke; CI, confidence interval.

10.7717/peerj.20745/supp-3 Supplemental Information 3 Characteristics of excluded and included patients. Abbreviations: CE, cardioembolism; EVT, endovascular therapy; IVT, intravenous thrombolysis; LAA, large artery atherosclerosis; NIHSS, National Institute of Health Stroke Scale; SAO, small artery occlusion; SOC, stroke of other determined cause; SUC, stroke of undetermined cause; TOAST, Trial of Org 10172 in Acute Stroke Treatment. *P <0.05.

10.7717/peerj.20745/supp-4 Supplemental Information 4 Baseline characteristics of eligible patients lost to follow-up *versus* eligible patients included in the analysis. Abbreviations: CE, cardioembolism; EVT, endovascular therapy; IVT, intravenous thrombolysis; LAA, large artery atherosclerosis; NIHSS, National Institute of Health Stroke Scale; SAO, small artery occlusion; SOC, stroke of other determined cause; SUC, stroke of undetermined cause; TOAST, Trial of Org 10172 in Acute Stroke Treatment. *P <0.05.

10.7717/peerj.20745/supp-5 Supplemental Information 5 Multivariable logistic regression for predicting poor functional outcome when TPM4 level as a dichotomous variable. ^a^ The TPM4 level is transformed into a dichotomous variable according to the optimal cuttoff value (2121.67ng/mL) calculated by the ROC curve before fitting the logistic regression model. Abbreviations: CI, confidence interval; EVT, endovascular therapy; IVT, intravenous thrombolysis; NIHSS, National Institute of Health Stroke Scale; NA, not applicable; OR, odds ratio; Ref, reference; TPM4, Tropomyosin 4. *P <0.05.

10.7717/peerj.20745/supp-6 Supplemental Information 6 Subgroup analysis of association between TPM4 level (as a continuous variable) and poor functional outcome. The presented ORs and 95% CIs are based on binary logistic regression analyses with adjustment for age, sex, National Institutes of Health Stroke Scale, atrial fibrillation, current smoking, alcohol consumption, the Trial of ORG 10172 in Acute Stroke Treatment classification and reperfusion therapy, except for the stratified variable. Abbreviations: Bridge treatment, intravenous thrombolysis and endovascular therapy; CI, Confidence interval; EVT, endovascular therapy; IVT, intravenous thrombolysis; OR, odds ratio; TPM4, Tropomyosin 4.

10.7717/peerj.20745/supp-7 Supplemental Information 7 STARD checklist. Standards for Reporting Diagnostic Accuracy Studies (STARD) checklist.

## Additional Information and Declarations

### Data Availability

The following information was supplied regarding data availability:

The raw data is available in the Supplemental Files.
