# Supplementary figures and images for: Admission serum tropomyosin 4 levels predict 1-year functional outcomes in acute ischemic stroke
Source: PeerJ. 2026 Feb 4;14:e20745. doi: 10.7717/peerj.20745 (PMC12882732; doi:10.7717/peerj.20745)

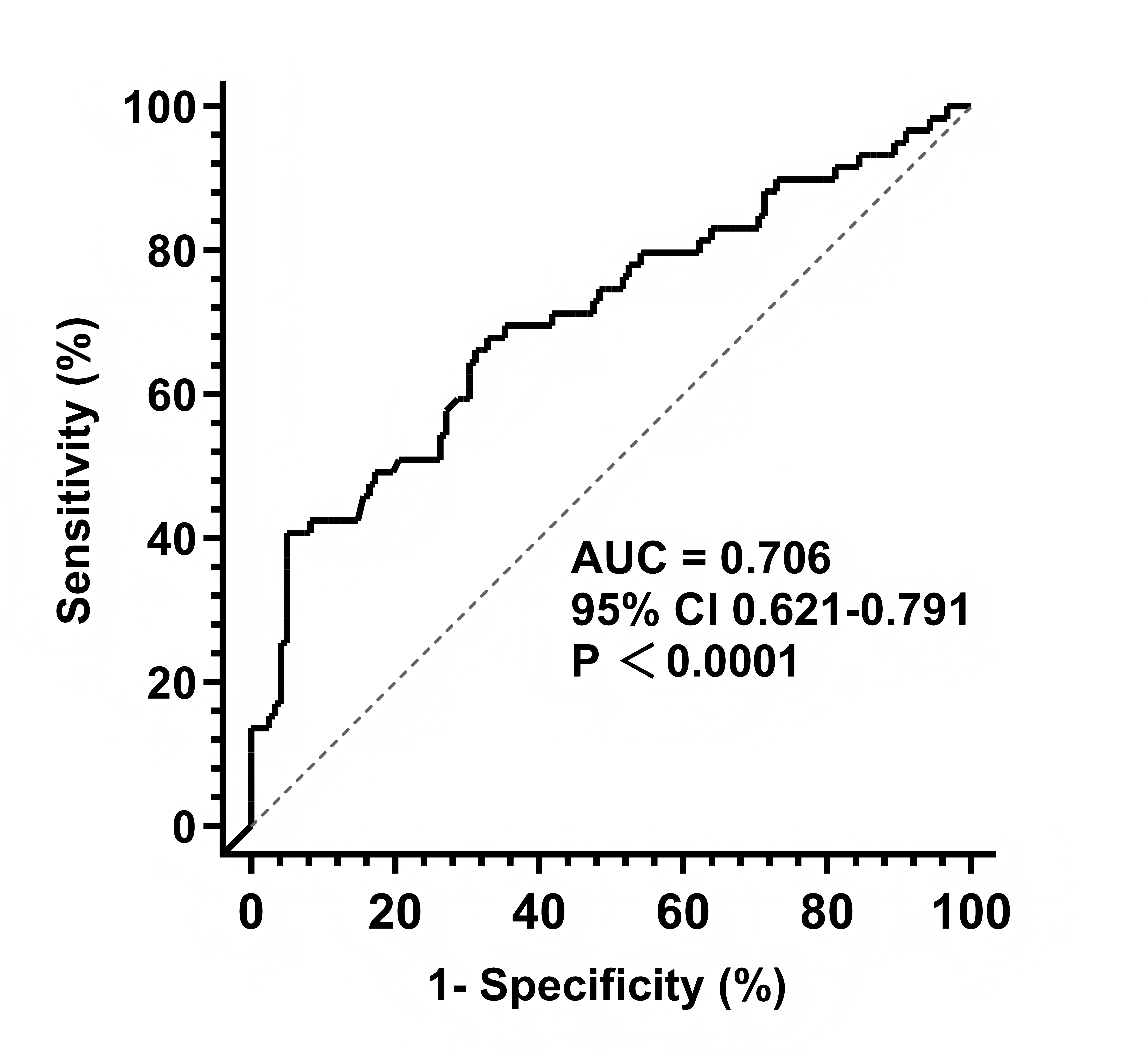

Supplement: Supplemental Information 2 — Abbreviations: AUC, area under the curve; AIS, acute ischemic stroke; CI, confidence interval. [file peerj-14-20745-s002.jpg]
