# Supplementary material for: Admission serum tropomyosin 4 levels predict 1-year functional outcomes in acute ischemic stroke
Source: PeerJ. 2026 Feb 4;14:e20745. doi: 10.7717/peerj.20745 (PMC12882732; doi:10.7717/peerj.20745)
Supplement: Supplemental Information 3 — Abbreviations: CE, cardioembolism; EVT, endovascular therapy; IVT, intravenous thrombolysis; LAA, large artery atherosclerosis; NIHSS, National Institute of Health Stroke Scale; SAO, small artery occlusion; SOC, stroke of other determined cause; SUC, stroke of undetermined cause; TOAST, Trial of Org 10172 in Acute Stroke Treatment. *P <0.05. [file peerj-14-20745-s003.docx]

**Table S1** Characteristics of excluded and included patients.

| Variables | Included (n=181) | Excluded (n=1182) | *P* value |
| --- | --- | --- | --- |
| Demographics |  |  |  |
| Age, y | 66 (55-72) | 67 (56-74) | 0.057 |
| Male | 116 (64.1) | 730 (61.8) | 0.548 |
| Clinical features |  |  |  |
| Onset-to-admission interval, hours | 5.0 (3.0-10.0) | 5.0 (3.0-13.3) | 0.023* |
| NIHSS | 8 (3-14) | 9 (3-15) | 0.159 |
| Vascular risk factors |  |  |  |
| Hypertension | 111 (61.3) | 708 (59.9) | 0.715 |
| Diabetes mellitus | 49 (27.1) | 348 (29.4) | 0.513 |
| Hyperlipidemia | 23 (12.7) | 166 (14.0) | 0.628 |
| Coronary artery disease | 20 (11.0) | 121 (10.2) | 0.738 |
| Atrial fibrillation | 54 (29.8) | 357 (30.2) | 0.920 |
| Stroke history | 19 (10.5) | 126 (10.7) | 0.947 |
| TOAST classification |  |  | 0.014* |
| LAA | 82 (45.3) | 527 (44.6) |  |
| CE | 54 (29.8) | 240 (20.3) |  |
| SAO | 28 (15.5) | 247 (20.9) |  |
| SOC | 5 (2.8) | 72 (6.1) |  |
| SUC | 12 (6.6) | 96 (8.1) |  |
| Reperfusion therapy |  |  | 0.093 |
| No | 104 (57.5) | 560 (47.4) |  |
| IVT | 20 (11.0) | 160 (13.5) |  |
| EVT | 46 (25.4) | 377 (31.9) |  |
| Bridge treatment | 11 (6.1) | 85 (7.2) |  |

Abbreviations: CE, cardioembolism; EVT, endovascular therapy; IVT, intravenous thrombolysis; LAA, large artery atherosclerosis; NIHSS, National Institute of Health Stroke Scale; SAO, small artery occlusion; SOC, stroke of other determined cause; SUC, stroke of undetermined cause; TOAST, Trial of Org 10172 in Acute Stroke Treatment.

**P* <0.05.
