# Supplementary material for: Admission serum tropomyosin 4 levels predict 1-year functional outcomes in acute ischemic stroke
Source: PeerJ. 2026 Feb 4;14:e20745. doi: 10.7717/peerj.20745 (PMC12882732; doi:10.7717/peerj.20745)
Supplement: Supplemental Information 4 — Abbreviations: CE, cardioembolism; EVT, endovascular therapy; IVT, intravenous thrombolysis; LAA, large artery atherosclerosis; NIHSS, National Institute of Health Stroke Scale; SAO, small artery occlusion; SOC, stroke of other determined cause; SUC, stroke of undetermined cause; TOAST, Trial of Org 10172 in Acute Stroke Treatment. *P <0.05. [file peerj-14-20745-s004.docx]

**Table S2** Baseline characteristics of eligible patients lost to follow-up versus eligible patients included in the analysis

| Variables | Included (n=181) | Lost to follow-up (n=48) | *P* value |
| --- | --- | --- | --- |
| Demographics |  |  |  |
| Age, y | 66 (55-72) | 67 (56-74) | 0.446 |
| Male | 116 (64.1) | 32 (66.7) | 0.740 |
| Clinical features |  |  |  |
| Onset-to-admission interval, hours | 5.0 (3.0-10.0) | 5 (3.0-8) | >0.999 |
| NIHSS | 8 (3-14) | 16 (4-20) | 0.002* |
| Vascular risk factors |  |  |  |
| Hypertension | 111 (61.3) | 30 (62.5) | 0.882 |
| Diabetes mellitus | 49 (27.1) | 12 (25.0) | 0.773 |
| Hyperlipidemia | 23 (12.7) | 7 (14.6) | 0.732 |
| Coronary artery disease | 20 (11.0) | 8 (16.7) | 0.291 |
| Atrial fibrillation | 54 (29.8) | 19 (39.6) | 0.198 |
| Stroke history | 19 (10.5) | 5 (10.4) | 0.987 |
| TOAST classification |  |  | 0.580 |
| LAA | 82 (45.3) | 27 (56.3) |  |
| CE | 54 (29.8) | 12 (25.0) |  |
| SAO | 28 (15.5) | 4 (8.3) |  |
| SOC | 5 (2.8) | 1(2.1) |  |
| SUC | 12 (6.6) | 4 (8.3) |  |
| Reperfusion therapy |  |  | 0.410 |
| No | 104 (57.5) | 21 (43.8) |  |
| IVT | 20 (11.0) | 7 (14.6) |  |
| EVT | 46 (25.4) | 16 (33.3) |  |
| Bridge treatment | 11 (6.1) | 4 (8.3) |  |

Abbreviations: CE, cardioembolism; EVT, endovascular therapy; IVT, intravenous thrombolysis; LAA, large artery atherosclerosis; NIHSS, National Institute of Health Stroke Scale; SAO, small artery occlusion; SOC, stroke of other determined cause; SUC, stroke of undetermined cause; TOAST, Trial of Org 10172 in Acute Stroke Treatment.

**P* <0.05.
