# Supplementary material for: Admission serum tropomyosin 4 levels predict 1-year functional outcomes in acute ischemic stroke
Source: PeerJ. 2026 Feb 4;14:e20745. doi: 10.7717/peerj.20745 (PMC12882732; doi:10.7717/peerj.20745)
Supplement: Supplemental Information 5 — a The TPM4 level is transformed into a dichotomous variable according to the optimal cuttoff value (2121.67ng/mL) calculated by the ROC curve before fitting the logistic regression model. Abbreviations: CI, confidence interval; EVT, endovascular therapy; IVT, intravenous thrombolysis; NIHSS, National Institute of Health Stroke Scale; NA, not applicable; OR, odds ratio; Ref, reference; TPM4, Tropomyosin 4. *P <0.05. [file peerj-14-20745-s005.docx]

**Table S3** Multivariable logistic regression for predicting poor functional outcome when TPM4 level as a dichotomous variable.

| Variables | Adjusted OR | 95% CI | *p* value |
| --- | --- | --- | --- |
| Age | 1.044 | 1.008-1.081 | 0.017 |
| NIHSS | 1.158 | 1.079-1.243 | <0.001* |
| Reperfusion therapy |  |  | 0.704 |
| No | NA | NA | Ref |
| IVT | 0.579 | 0.138-2.436 | 0.456 |
| EVT | 1.285 | 0.501-3.298 | 0.602 |
| Bridge treatment | 0.665 | 0.127-3.475 | 0.629 |
| Dichotomous TPM4^a^ | 0.115 | 0.039-0.338 | <0.001* |

^a^The TPM4 level is transformed into a dichotomous variable according to the optimal cuttoff value (2121.67ng/mL) calculated by the ROC curve before fitting the logistic regression model.

Abbreviations: CI, confidence interval; EVT, endovascular therapy; IVT, intravenous thrombolysis; NIHSS, National Institute of Health Stroke Scale; NA, not applicable; OR, odds ratio; Ref, reference; TPM4, Tropomyosin 4.

**P* <0.05.
