# Supplementary material for: Admission serum tropomyosin 4 levels predict 1-year functional outcomes in acute ischemic stroke
Source: PeerJ. 2026 Feb 4;14:e20745. doi: 10.7717/peerj.20745 (PMC12882732; doi:10.7717/peerj.20745)
Supplement: Supplemental Information 6 — The presented ORs and 95% CIs are based on binary logistic regression analyses with adjustment for age, sex, National Institutes of Health Stroke Scale, atrial fibrillation, current smoking, alcohol consumption, the Trial of ORG 10172 in Acute Stroke Treatment classification and reperfusion therapy, except for the stratified variable. Abbreviations: Bridge treatment, intravenous thrombolysis and endovascular therapy; CI, Confidence interval; EVT, endovascular therapy; IVT, intravenous thrombolysis; OR, odds ratio; TPM4, Tropomyosin 4. [file peerj-14-20745-s006.docx]

**Table S4** Subgroup analysis of association between TPM4 level (as a continuous variable) and poor functional outcome.

| Subgroup | Event/Total | OR (95% CI) | P interaction |
| --- | --- | --- | --- |
| Age, year |  |  | 0.511 |
| ＜65 | 16/86 (18.6) | 0.005 (0.000-0.856) |  |
| ≥65 | 43/95 (45.26) | 0.008 (0.000-0.286) |  |
| Sex |  |  | 0.071 |
| Male | 30/116 (25.9) | 0.003 (0.000-0.113) |  |
| Female | 29/65 (44.6) | 0.030 (0.000-2.919) |  |
| NIHSS |  |  | 0.181 |
| ≤5 | 5/74 (6.8) | 0.087 (0.000-5.581) |  |
| ＞5 | 54/107 (50.5) | 0.044 (0.004-0.526) |  |
| Atrial fibrillation |  |  | 0.710 |
| No | 29/127 (22.8) | 0.017 (0.001-0.389) |  |
| Yes | 30/54 (55.6) | 0.047 (0.001-2.042) |  |
| TOAST classification |  |  | 0.868 |
| LAA | 26/82 (31.7) | 0.013 (0.000-0.434) |  |
| Non-LAA | 33/99 (33.3) | 0.044 (0.002-1.224) |  |
| Reperfusion therapy |  |  | 0.499 |
| No | 28/104 (26.9) | 0.009 (0.000-0.351) |  |
| Yes | 31/77 (40.3) | 0.036 (0.001-0.922) |  |
| IVT | 5/20 (25.0) | NA |  |
| EVT/ Bridge treatment | 26/57 (45.6) | 0.021 (0.000-0.936) |  |
| EVT | 22/46 (47.8) | 0.102 (0.002-5.182) |  |
| Bridge treatment | 4/11(36.4) | NA |  |

The presented ORs and 95% CIs are based on binary logistic regression analyses with adjustment for age, sex, National Institutes of Health Stroke Scale, atrial fibrillation, current smoking, alcohol consumption, the Trial of ORG 10172 in Acute Stroke Treatment classification and reperfusion therapy, except for the stratified variable.

Abbreviations: Bridge treatment, intravenous thrombolysis and endovascular therapy; CI, Confidence interval; EVT, endovascular therapy; IVT, intravenous thrombolysis; OR, odds ratio; TPM4, Tropomyosin 4.
